# Solvent-free sonochemistry: Sonochemical organic synthesis in the absence of a liquid medium

**DOI:** 10.3762/bjoc.13.179

**Published:** 2017-09-04

**Authors:** Deborah E Crawford

**Affiliations:** 1 School of Chemistry and Chemical Engineering, Queen’s University Belfast, David Keir Building, 39–123 Stranmillis Road, Belfast, BT9 5AG, Northern Ireland, UK

**Keywords:** mechanochemistry, organic, solvent-free, sonochemistry, synthesis

## Abstract

Sonochemistry, i.e., the application of mechanical energy in the form of sound waves, has recently been recognised for its similarity to mechanochemistry and is now included under the umbrella term of mechanochemistry. Typically, due to the hypothesised cavitation mechanism, a liquid medium is considered as a necessity for a process to take place as a result of ultrasonic irradiation. In view of this, condensation reactions between solid reagents in the complete absence of solvent were carried out successfully by ultrasonic irradiation with the importance of particle size being highlighted. This work increases the potential of sonochemistry in the drive towards a sustainable future.

## Introduction

**Figure 1:**
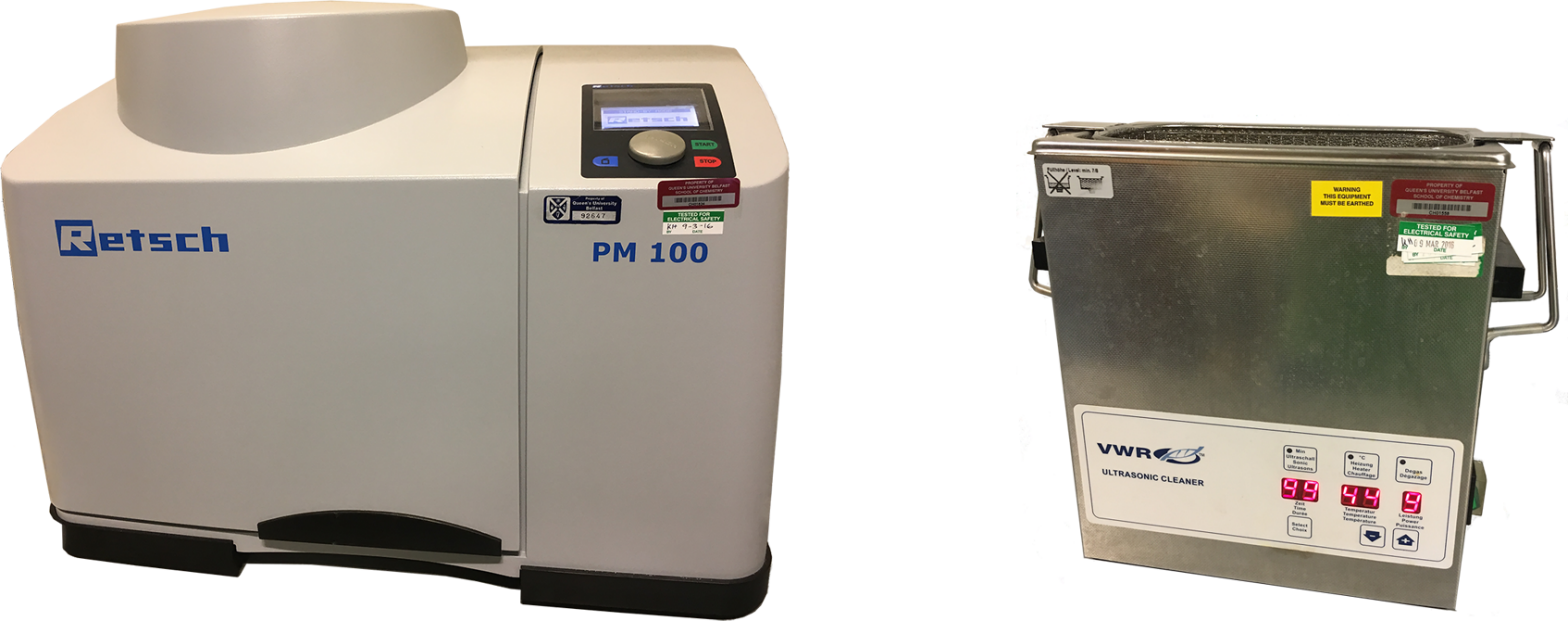
Typical laboratory employed planetary ball mill and ultrasonic bath.

Mechanochemistry is typically regarded as the grinding of solid reagents in a ball mill (or mortar and pestle), to instigate and accelerate chemical reactions [1]. In recent years, mechanochemistry has evolved to include techniques such as shearing [2], microfluidics [3] and twin screw extrusion [4–6]. More recently, sonochemistry has been included under the umbrella term of mechanochemistry [7] as it has demonstrated excellent potential when instigating chemical activity in solutions by applying mechanical energy (Figure 1) [8–9].

Sonochemistry is hypothesised to originate from acoustic cavitation and bubble collapse as a result of the mechanical effects of sounds on liquids [8–9]. Bubble collapse in particular results in intense compressional heating, thereby creating hot spots, a phenomenon currently employed to explain the processes occurring in ball milling [7]. It must also be noted that there is a similar technology available to sonochemistry, that is considered to be less harsh than mechanochemistry, and this is resonant acoustic mixing (RAM). The RAM mixes by controlling the vibration applied to the material through acceleration and frequency, and therefore is actually mechanistically different from sonochemical mixing [8–9]. Other effects that have been found to be common for both sonochemistry and mechanochemistry include: local heating, crystal deformation and phase transitions amongst others [7].

Ultrasonic irradiation is commonly carried out on liquid/gas mixtures (for gas removal), liquid/liquid mixtures and liquid/solid mixtures. The technique is used extensively in materials chemistry, for example, it has been demonstrated to be one of the most efficient methods to exfoliate layered materials such as graphite (to form graphene) [10], but it has also been employed in the formation of organometallic [11] and organic compounds [12]. Great success has been found in the treatment of waste water by ultrasonic irradiation, to remove heavy metals or degrade aromatic constituents [13].

Metal catalysts are prepared by the sonication of metal halides (e.g., Pt and Pd – reduction of metal) in the presence of Li and THF [14–15]. Furthermore, the catalytic behaviour of catalysts such as Raney Nickel, has reportedly been increased solely due to the effect of using ultrasound [16]. Catalyst coatings, such as metal oxide, can be broken up and removed as a result of ultrasonic cavitation, therefore this technology overcomes the drawbacks of reacting a solid and a liquid in a heterogeneous system, allowing the reaction to proceed further [16].

The reaction of toluene with benzyl bromide in the presence of KCN/Al_2_O_3_, is an example of organic synthesis employing ultrasound [17]. Interestingly, the conventional solution method results in the alkylation of the toluene aromatic ring, however, when sonication is employed, a reaction between benzyl bromide and KCN occurs producing PhCH_2_CN, indicating that alternative products can be formed using this technique, as with ball milling.

The Knoevenagel condensation [18], Michael addition [19] and Biginelli reactions [20] amongst others have been instigated by ultrasonic irradiation in the presence of solvents such as pyridine and methanol, resulting in a decrease of their reaction times from >10 hours to 1–2 hours. Also, in some cases sonication greatly improved the yield, for example in a Vilsmeier–Haack reaction [21]; in addition selectivity can also be improved as demonstrated in a Pinacol coupling whereby a *meso*-isomer was the dominant product, a result only observed when the reaction is sonicated [22].

## Results and Discussion

The presence of a liquid medium in a system undergoing ultrasonic irradiation is greatly important to facilitate the cavitation process and a consequence of this is that there has not been any research into sonochemical reactions being carried out in the absence of solvent, or a liquid reagent [8–9]. Herein, we report two condensation reactions (investigated extensively by ball milling), one to form salen ligand **1** by sonicating *o*-vanillin and 1,2-phenylenediamine, and the second to form 1,3-indandione **2** from ninhydrin and dimedone. Both systems were investigated in the complete absence of solvent and without the presence of any grinding media (such as inert silica beads) to help mediate the reaction. The aldol reaction was successfully carried out by twin screw extrusion, as I have reported previously [6]. The success of both of these reactions by ultrasound irradiation in the absence of solvent creates potential for organic synthesis to be carried out by applying a milder form of mechanical energy, i.e., sound waves. As a result, it may be possible that reactions which are particularly sensitive to intense mechanical energy (and may undergo degradation) may be successful by ultrasonic irradiation.

**Scheme 1:**
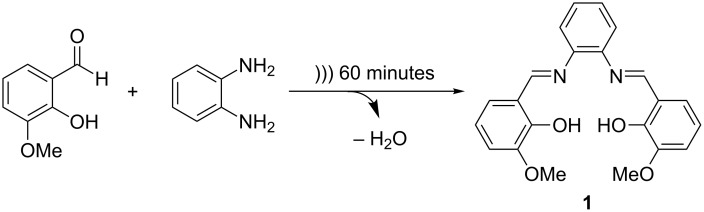
Reaction between *o*-vanillin and 1,2-phenylenediamine by ultrasonic irradiation for 60 minutes.

**Figure 2:**
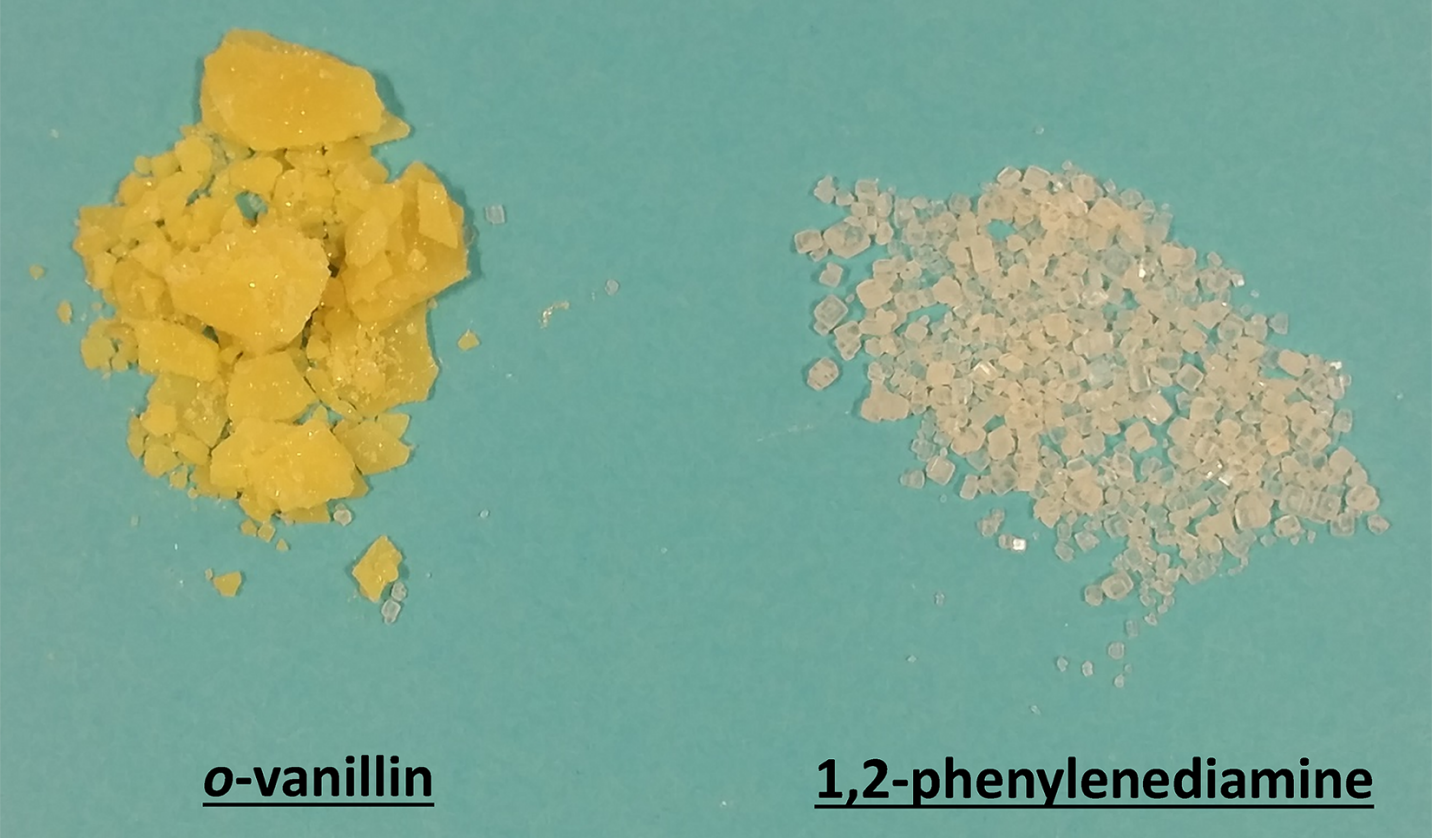
*o-*Vanillin in its flake form and 1,2-phenylenediamine in its bead form.

It must be noted that the conventional reaction between *o-*vanillin and 1,2-phenylenediamine requires refluxing for 9 hours in ethanol for a complete conversion to the product. For the initial sonochemistry experiments (Scheme 1), both reagents were used as received, *o-*vanillin came in the form of small flakes and 1,2-phenylenediamine was received as large crystalline beads (Figure 2).

Upon sonication of the mixed reagents (using a standard ultrasonication bath with a frequency of 35 kHz) for 60 minutes, it was observed that the temperature of the ultrasonic bath increased to 70–75 °C, causing both reagents to form a melt (*o*-vanillin has a melting point of 42 °C, 1,2-phenylenediamine has a melting point of 104 °C), which is likely to be the result of an eutectic melt forming. This was quite surprising as the melting point of 1,2-phenylenediamine is greater than that of the observed temperature of the ultrasonic bath. The molten substance then changed to a hard solid form and not the preferred free flowing solid. It was expected that because the reagents melted then they would have reacted completely to form the product, aided by the help of heating. However, ^1^H NMR spectroscopy showed that the conversion to the product was only 36%, therefore, the reaction did not proceed significantly as a result of the high temperature, and the reaction was potentially hindered as a result of the hard solid formed.

**Figure 3:**
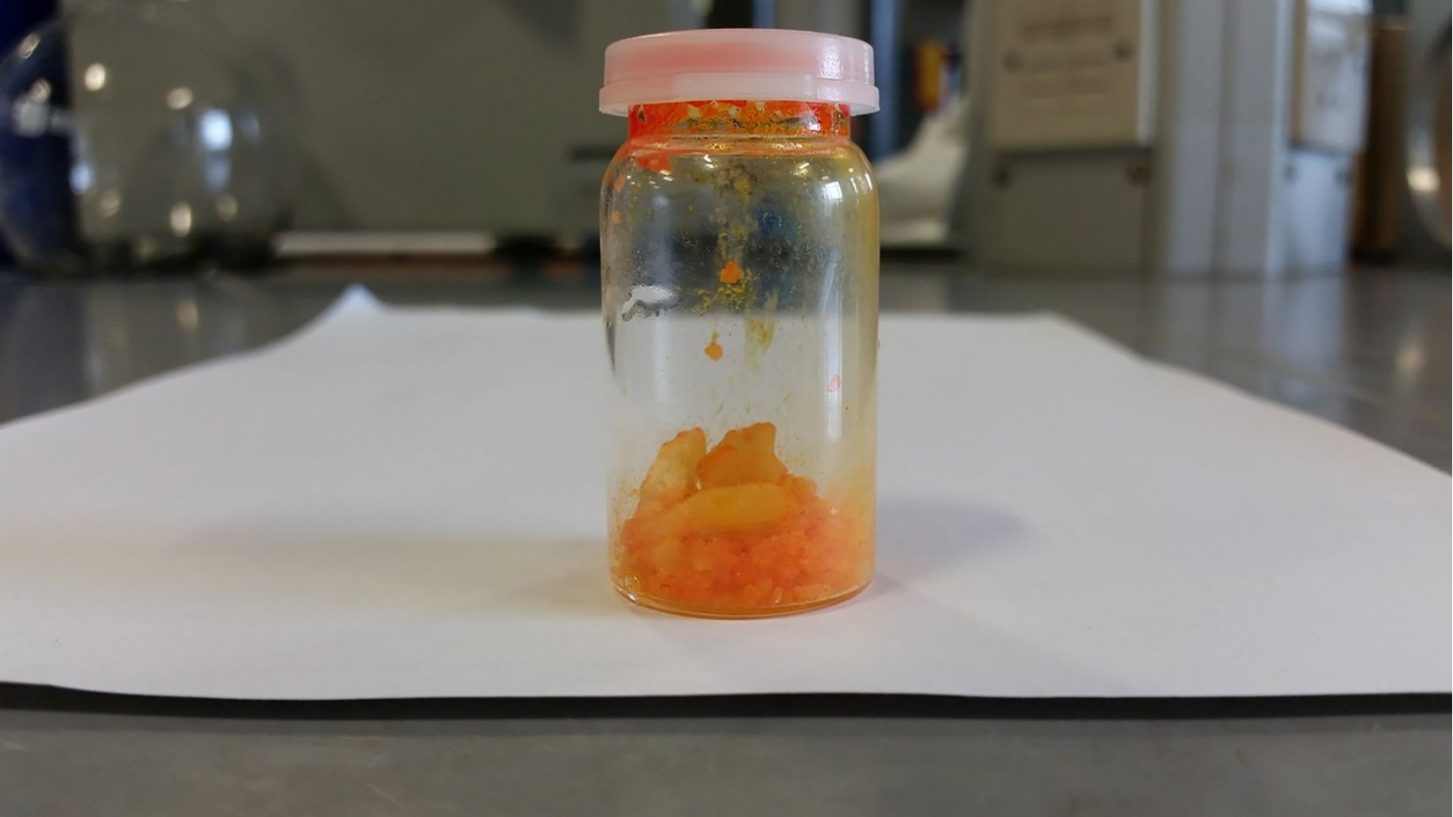
Clear separation of the reagents observed, with orange coated beads of 1,2-phenylenediamine residing at the bottom of the mixture.

Stopping the reaction to grind this solid form into a free flowing solid would lead to inaccurate results as mechanical energy in the form of grinding could have a significant effect on the outcome of the reaction. Therefore, as the application of heat may have an effect on the conversion to product and the mixture needed to remain as a free slowing solid, sonication was carried out for 10 minute intervals, preventing an increase in temperature and melting of the reagents (alternatively a cooling fluid could be used). After 60 minutes of sonicating, a colour change was observed (Figure 3 – to bright orange) but there was a clear separation between the two solids, indicating that the variation of particle size and morphology was too great for the reaction to proceed quantitatively.

Therefore, both reagents were ground and sieved to both be fine powders of particle size <500 µm. A ca. 0.2 g mixture was sonicated for 60 minutes (keeping the temperature of the bath at ambient temperature) and it was clear to see that a more successful reaction had taken place. A homogeneous orange solid was produced; however, there was an increase in the pressure of the system that was too great to be withheld in the 2 mL vial employed. This was presumably due to the production of the byproduct – water, which was seen to be in its vapour form, most likely due to the heat produced from the exothermic reaction of the aldehyde and the diamine. This indicated that a greater free volume (also known as ‘headspace’) in the vial was required to accommodate this increase in pressure.

A larger vial (25 mL) was then employed, which was able to sustain the pressure of the water vapour produced in the system, and with that there was a 5-fold scale-up of the reaction mixture from ca. 0.2 g to ca. 1.0 g. After 60 minutes of ultrasonic irradiation a bright orange free flowing solid was produced indicating that a reaction had occurred. ^1^H NMR spectroscopy showed that indeed a reaction had taken place to form the desired imine; however, a conversion to product of only 69% was determined. The experiment was repeated but ultrasonic irradiation was carried out for 90 minutes, leading to a marginally higher conversion to product of 73%.

**Figure 4:**
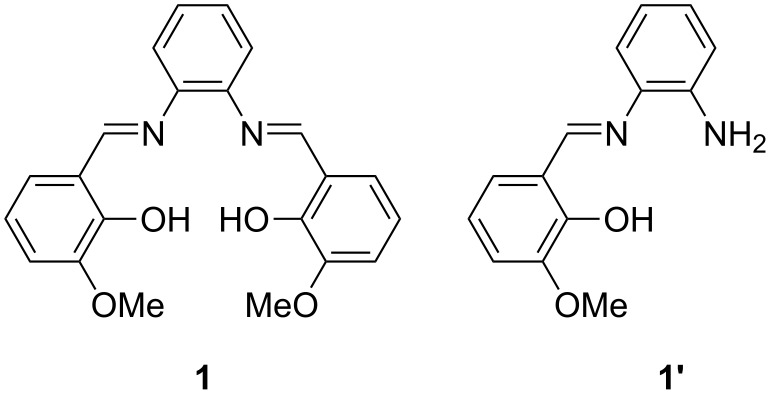
Chemical structures of the products obtained from the reaction between *o-*vanillin and 1,2-phenylenediamine.

In order to improve the rate of conversion to product, a mild base, anhydrous Na_2_CO_3_ (0.1 equiv) was added to the reaction. *o*-Vanillin, 1,2-phenylenediamine and Na_2_CO_3_ were then sonicated for 60 minutes to produce a dark red solid (similar to that obtained from solution). ^1^H NMR spectroscopy indicated that the reagents had almost all been consumed; however, it was noted that the ^1^H NMR spectrum was more complicated than expected with two peaks representing imine protons. It was determined that the desired product had formed along with the product from the 1:1 reaction of the aldehyde and the diamine, **1’** (Figure 4). Excess aldehyde was expected to be present; however, this was not the case (<1% present), indicating that the reaction mixture was still not completely homogeneous.

**Figure 5:**
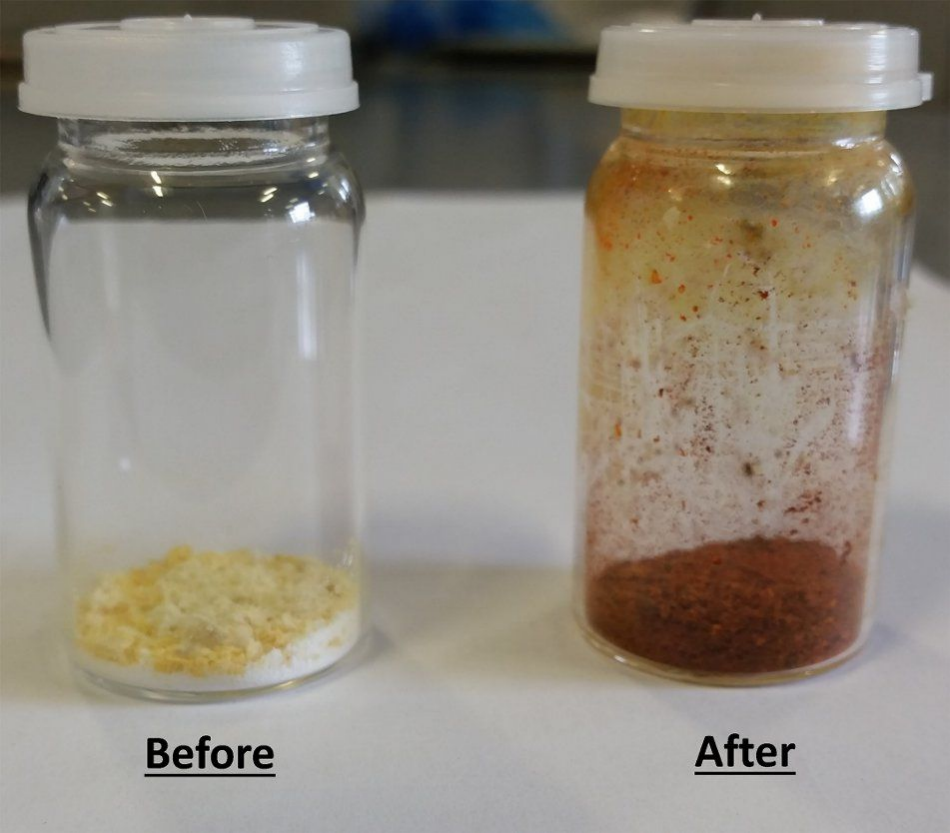
Reaction mixture before and after ultrasonic irradiation for 60 minutes.

**Figure 6:**
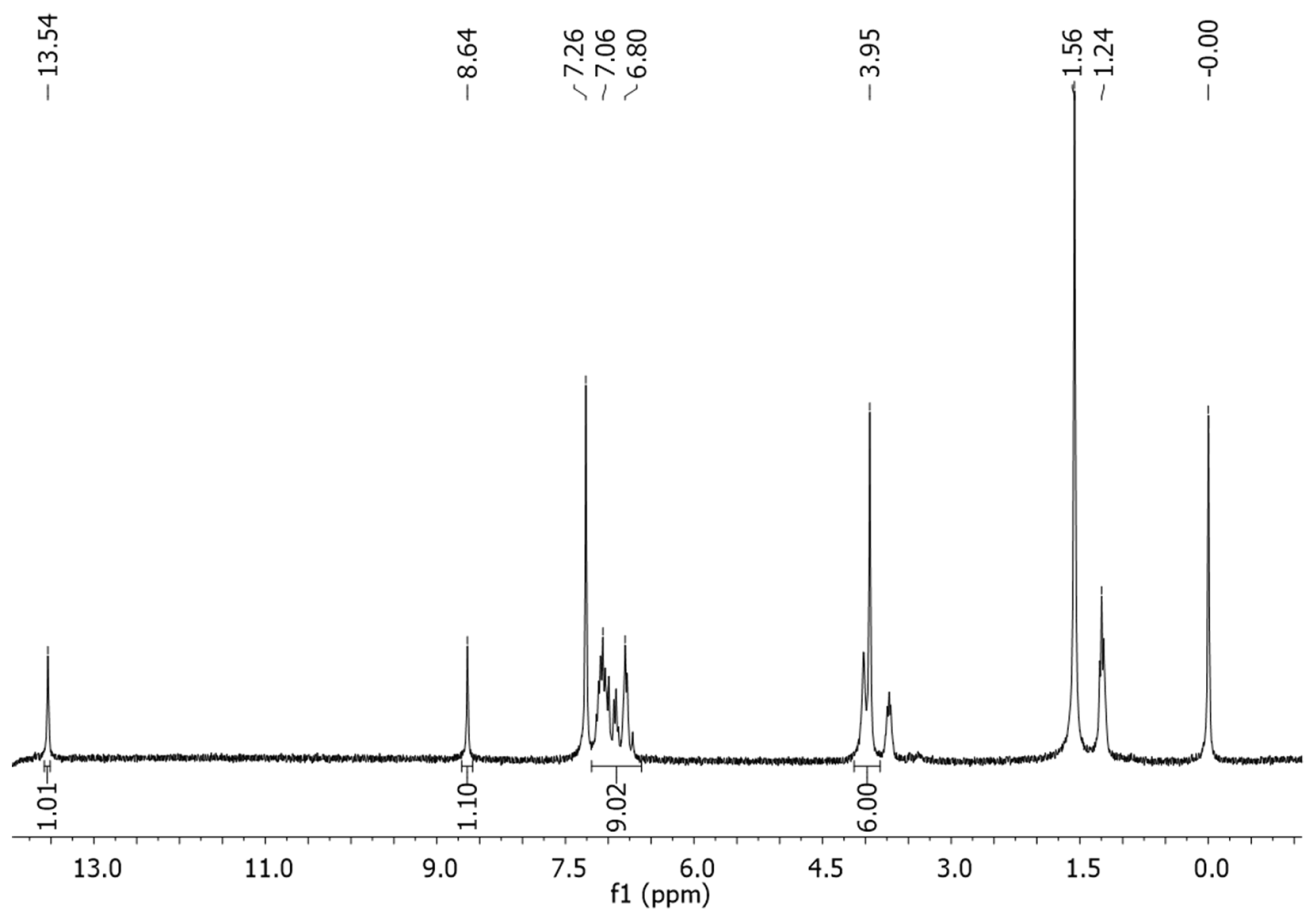
^1^H NMR spectrum of diimine **1** in CDCl_3_/EtOD.

In order to overcome this problem, the particle size of both starting materials was reduced further to <200 µm and sonicated for 60 minutes, resulting in a dark red powder (Figure 5). ^1^H NMR spectroscopy showed that the reaction had fully converted to the desired product – the desired diimine, **1** (Figure 6). Powder X-ray diffraction (PXRD) analysis shows that the powder patterns of both the sonochemical product and the solution product are the same and IR spectroscopy confirmed that an imine bond was present in the product, with no indication of an aldehyde functionality being present (see Supporting Information File 1).

Sonochemical reactions are reportedly irreproducible [11] and to determine if this was the case in the reaction between *o-*vanillin and 1,2-phenylenediamine, the experiment was repeated under the optimised conditions three times. In each case, the reaction was reproducible showing a complete conversion to the product (as determined by ^1^H NMR spectroscopy). In addition, a control reaction was carried out whereby the reagents (in the same concentrations) were mixed manually and left for several days without being agitated. Although there was some colour change in the mixture, the reaction had not proceeded significantly (after 3 days, <5% conversion to product was observed). Furthermore, the reaction was monitored in CDCl_3_ to ensure that the reactions were not proceeding as a result of exposure to the NMR spectroscopy solvent, which was found to be the case. Therefore, it is ultrasonic irradiation at room temperature that is instigating and accelerating this chemical reaction.

**Scheme 2:**
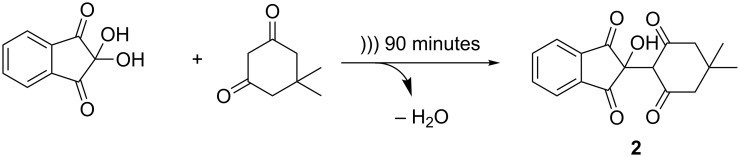
Aldol reaction between ninhydrin and dimedone to form **2**.

**Figure 7:**
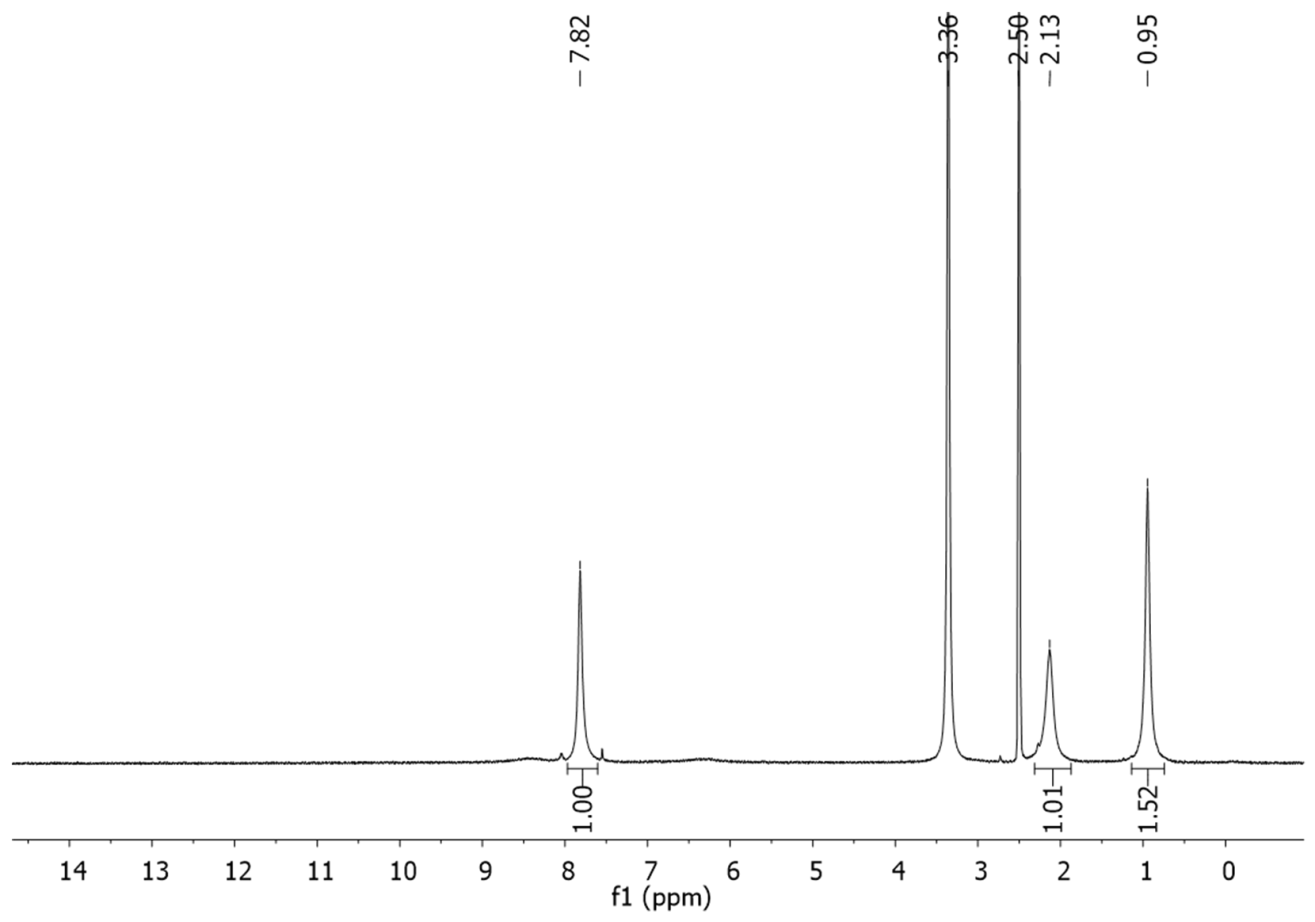
^1^H NMR spectrum of 1,3-indandione **2** in DMSO-*d*_6_.

Further confirmation that sonochemistry is a viable method to carry out solid state organic synthesis was obtained by carrying out an aldol reaction between ninhydrin and dimedone (Scheme 2). The optimised parameters from the previous system were applied, i.e., the particle size of the reagents was reduced (to <200 µm) and ultrasonic irradiation was carried out in 10 minute intervals to prevent melting of the reagents. After 90 minutes of sonicating, a pink solid was produced with ^1^H NMR spectroscopy indicating that a complete reaction had taken place between a hydroxy group of ninhydrin and the activated methylene of dimedone (Figure 7). This reaction has previously been carried out by twin screw extrusion in the absence of solvent [6], and it was confirmed that the same product was obtained by both synthetic methods (see Supporting Information File 1). A control experiment was carried out, whereby the reagents were mixed as two solids and left under ambient temperature and pressure for several weeks, after three weeks, a conversion of 72% to the desired product was observed, confirming the advantage of employing sonochemistry.

## Conclusion

In conclusion, the first examples of ultrasound induced solvent-free condensation reactions are reported, forming a Schiff base **1** (which has significant applications in catalysis) and a 1,3-indandione **2**. It was concluded that one of the key parameters in these reactions was the particle size of the starting materials, with a reduced particle size of <200 µm resulting in a homogeneous mixture leading to complete conversion to the product. This provides an excellent foundation for further investigations into solvent-free or solid-state sonochemistry, including studying a larger scope of chemical reactions and the mechanism behind which the liquid/solvent-free reactions occur. It also provides a means of applying a gentler form of mechanical energy to a system which may increase the range of organic and inorganic mechanochemical transformations that can be carried out (where grinding results in degradation of the material). Finally, as with ball milling, there is potential for the scale-up of sonochemical reactions, therefore aiding in the drive towards sustainable chemistry.

## Supporting Information

File 1 Experimental part.
