# Supplementary material for: Solvent-free sonochemistry: Sonochemical organic synthesis in the absence of a liquid medium
Source: Beilstein J Org Chem. 2017 Sep 4;13:1850–6. doi: 10.3762/bjoc.13.179 (PMC5629407; doi:10.3762/bjoc.13.179)
Supplement: File 1 — Experimental part. [file Beilstein_J_Org_Chem-13-1850-s001.pdf]

Supporting Information

for

Solvent-free sonochemistry: Sonochemical organic synthesis in the absence of a liquid medium

Deborah E. Crawford*

Address: School of Chemistry and Chemical Engineering, Queen’s University Belfast, David Keir Building, 39–123 Stranmillis Road, Belfast, BT9 5AG, Northern Ireland, UK

Email: Deborah E. Crawford - d.crawford@qub.ac.uk

*Corresponding author

Experimental part

1. Experimental details

All materials were purchased from Sigma-Aldrich UK with >98% purity. The particle size of the reagents was reduced by ball milling each reagent individually, in a 25 cm^3 stainless steel ball mill jar, with a 13.6 g ball bearing, at 25 Hz for 5 minutes (<500 µm) or 10 minutes (<200 µm) (employing a Retsch MM400 vibrational ball mill). The obtained powders were then sieved to obtain the desired particle size. Ultrasonic irradiation was carried out using a standard 35 kHz ultrasound bath. NMR spectroscopy was carried out with a Bruker Advance DPX 300 using $CDCl_3$ or DMSO-d_6 as solvent. Infrared spectroscopy was carried out using a Fourier Transform IR, Perkin Elmer Spectrum 1, with CsI beam splitter - 4000–200 cm^{-1} using a pressed KBr disc. X-ray powder diffraction was carried out using a PANanalytical X'Pert Pro diffractometer with Cu Kα radiation.

2. Synthesis of 1

O-Vanillin of a particle size <200 µm (0.50 g, 3.29 mmol), 1,2-phenylenediamine of particle size <200 µm (0.36 g, 3.29 mmol) and anhydrous Na_2CO_3 (0.035 g, 0.329 mmol) were mixed in a 25 mL glass vial. The mixture was sonicated at 35 kHz for 10 minutes and then the ultrasonic bath was left to cool back to 25 °C (from ca. 30 °C). This process was repeated six times so that the sample was irradiated by ultrasound for 60 minutes. This produced a dark red solid. ^1H NMR spectroscopy was carried out in $CDCl_3$.

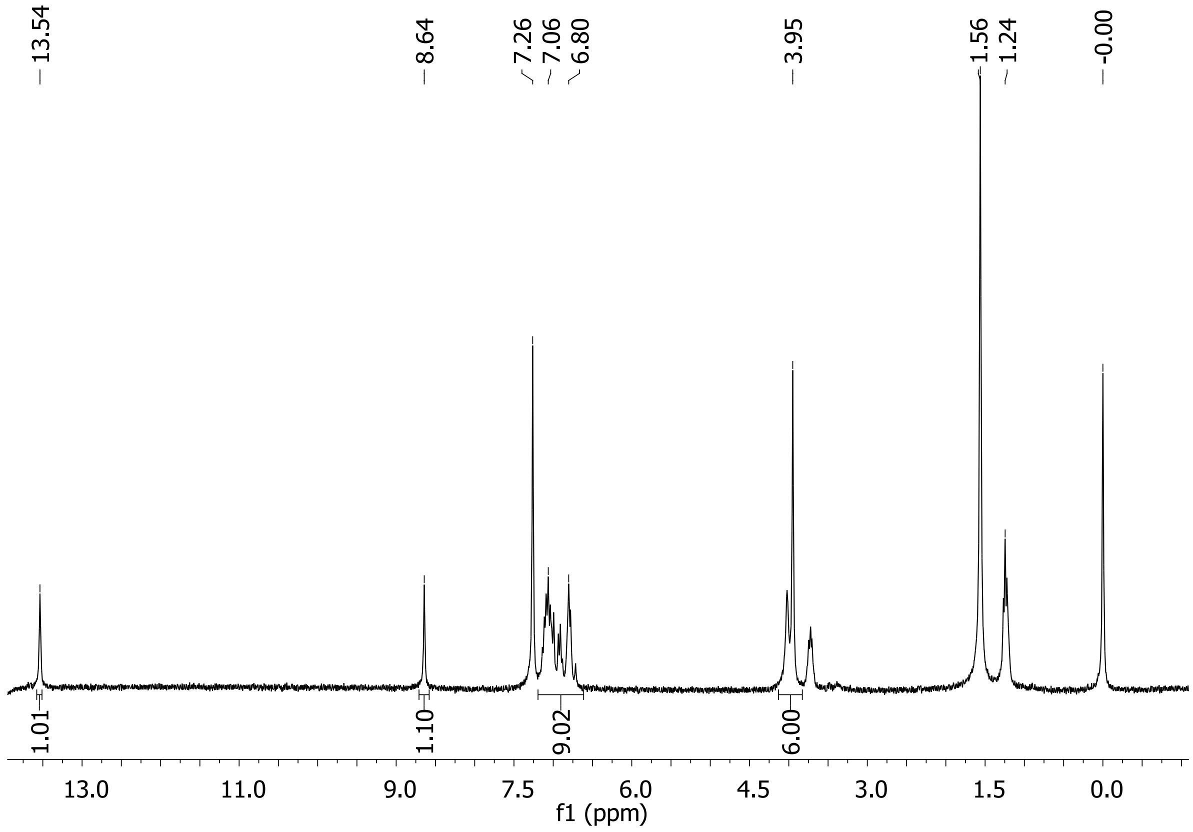

Figure S1: ^1H NMR spectrum of **1** in $CDCl_3$/EtOD.

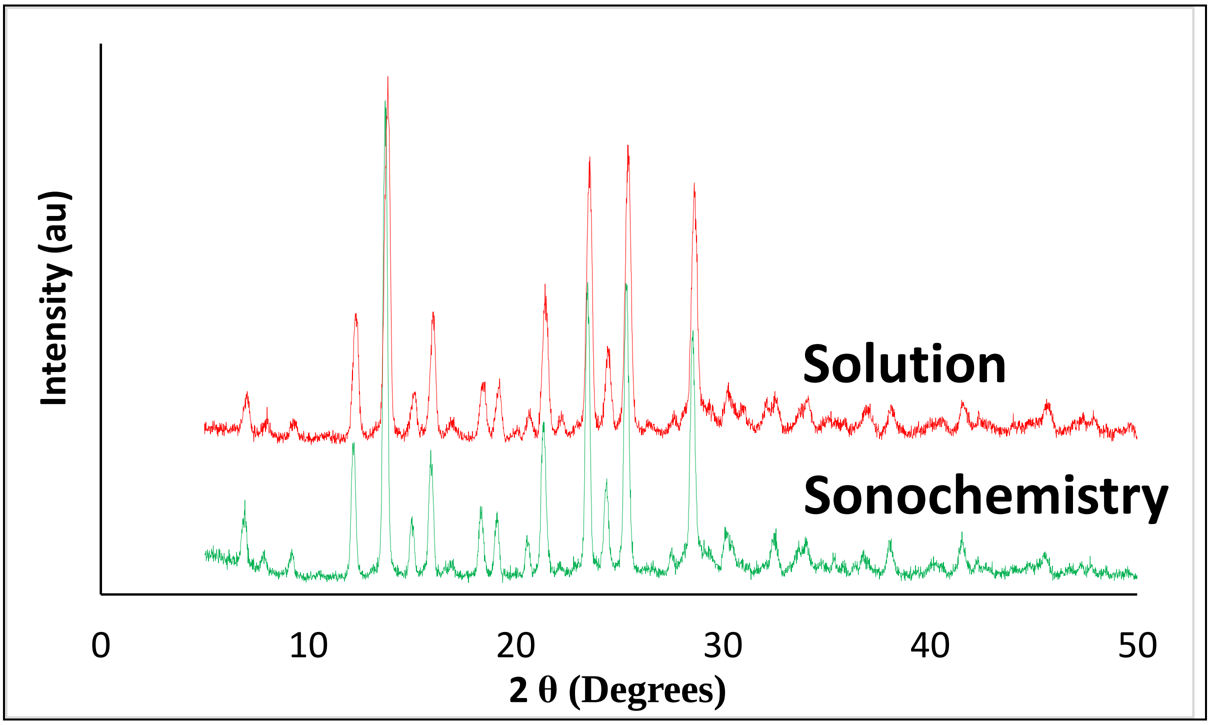

Figure S2: PXRD patterns of **1** prepared from solution and ultrasonication.

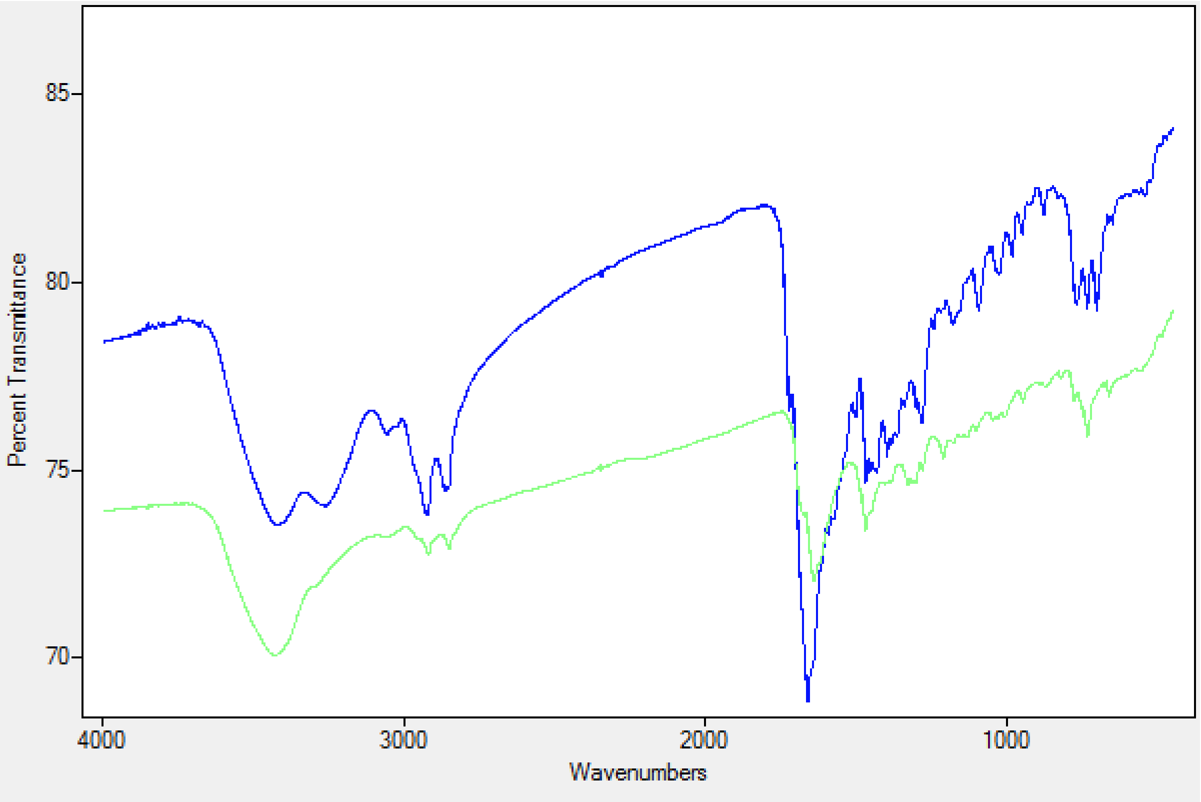

Figure S3: IR spectra of **1** (green – sonication, blue – solution) with an imine stretching band at 1645 cm^{-1} (with baseline corrected).

3. Synthesis of 2

Ninhydrin of a particle size <200 µm (0.50 g, 2.81 mmol) and dimedone of particle size < 200 µm (0.39 g, 2.81 mmol) were mixed in a 25 mL glass vial. The mixture was sonicated at 35 kHz for 10 minutes and then the ultrasonic bath was left to cool back to 25 °C (from ca. 30 °C). This process was repeated nine times so that the sample was irradiated by ultrasound for 90 minutes. This produced a pink solid. ^{1}H NMR spectroscopy was carried out in DMSO-d_6.

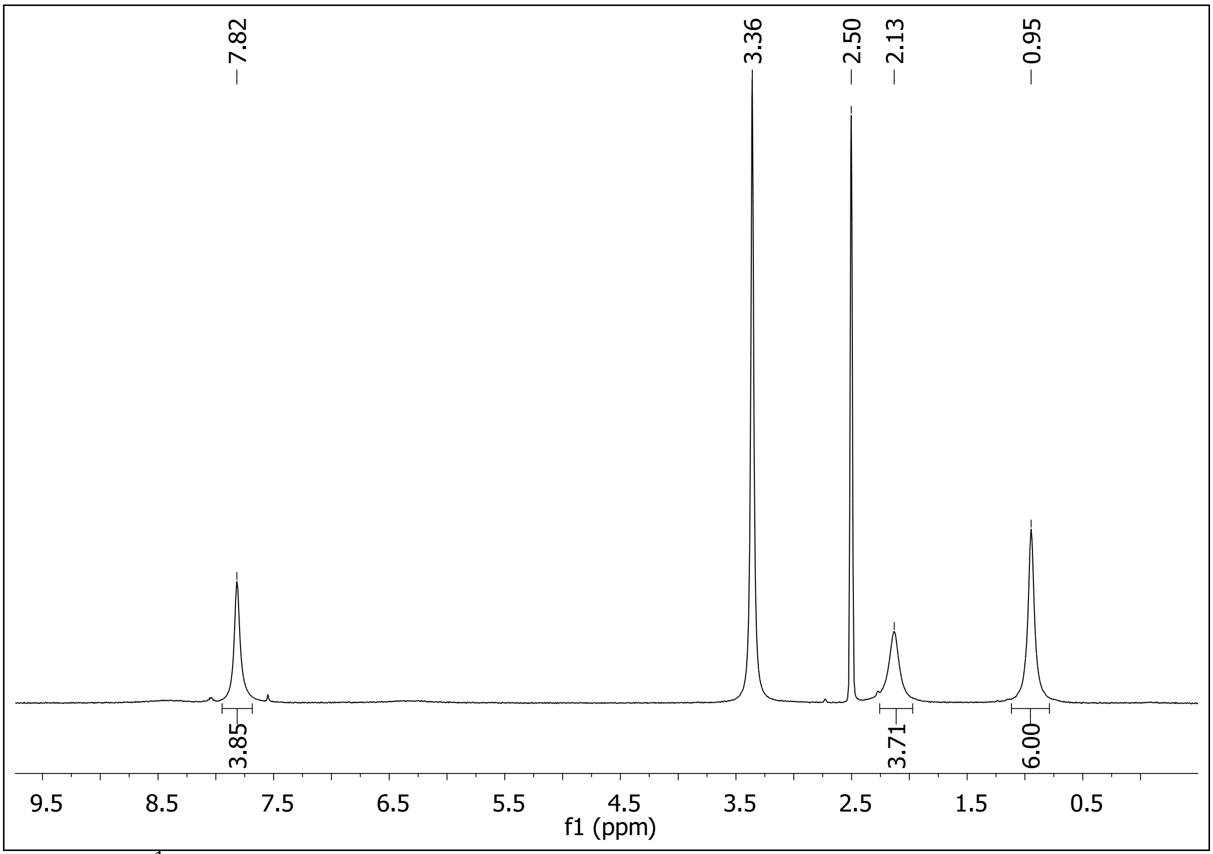

Figure S4: ^{1}H NMR spectrum of **2** in DMSO-d_6.

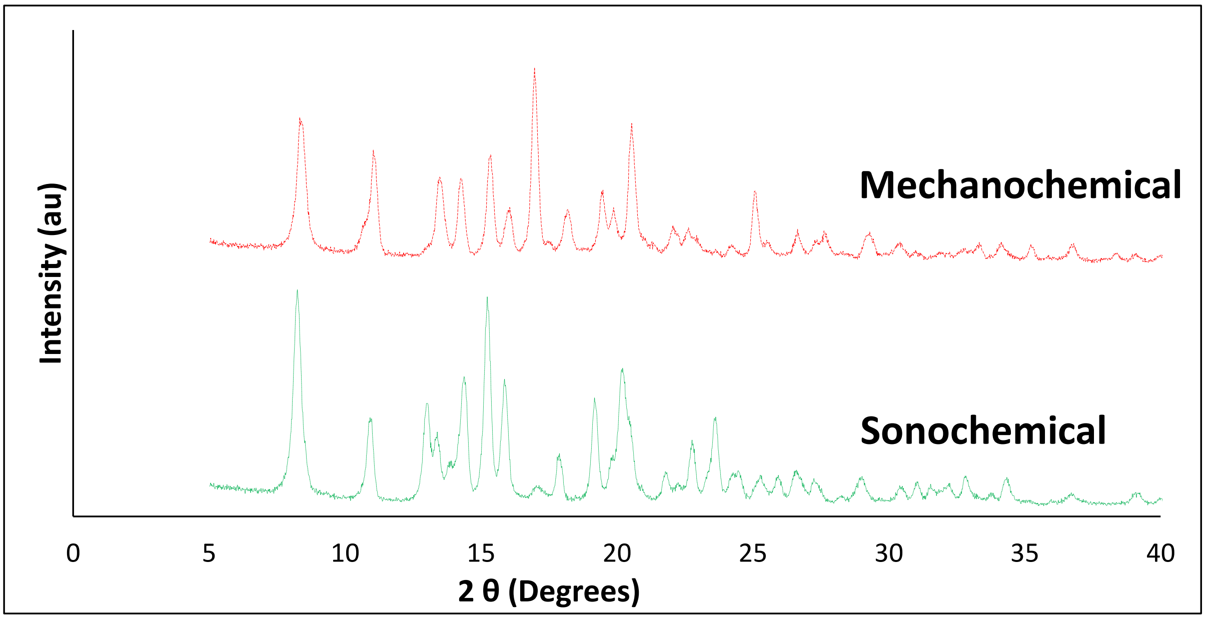

Figure S5: PXRD patterns of **2** prepared from twin screw extrusion and ultrasonication.
